# Eco-evolutionary dynamics and environmental detoxification jointly shape bacterial community response to antibiotic perturbation

**DOI:** 10.1093/ismejo/wrag248

**Published:** 2026-09-16

**Authors:** Johannes Cairns, Niina Smolander, Sanna Pausio, Olli Pitkänen, Meri Lindqvist, Manu Tamminen, Rishi Das Roy, Ville-Petri Friman, Lutz Becks, Ville Mustonen, Teppo Hiltunen

**Affiliations:** Department of Experimental Medical Science, Lund University, 223 62 Lund, Sweden; Science for Life Laboratory (SciLifeLab), Lund University, 223 62 Lund, Sweden; Department of Biology, University of Turku, Turku 20014, Finland; Turku Collegium for Science, Medicine and Technology, University of Turku, Turku 20014, Finland; Department of Computer Science, Department of Organismal and Evolutionary Biology, University of Helsinki, Helsinki 00014, Finland; Department of Biology, University of Turku, Turku 20014, Finland; Department of Biology, University of Turku, Turku 20014, Finland; Department of Biology, University of Turku, Turku 20014, Finland; Department of Biology, University of Turku, Turku 20014, Finland; Department of Biology, University of Turku, Turku 20014, Finland; HiLIFE, Institute of Biotechnology, University of Helsinki, Helsinki 00014, Finland; Department of Microbiology, University of Helsinki, Helsinki 00014, Finland; Department of Biology, University of Konstanz, Konstanz 78464, Germany; Department of Computer Science, Department of Organismal and Evolutionary Biology, University of Helsinki, Helsinki 00014, Finland; Department of Biology, University of Turku, Turku 20014, Finland

**Keywords:** microbial community resilience, priming effects, ampicillin resistance, antibiotic degradation, eco-evolutionary feedbacks

## Abstract

Microbial communities frequently encounter recurrent antibiotic disturbance, yet how ecological, evolutionary, and environmental processes jointly shape community responses remains unresolved. Here, we use priming to describe the history-dependent effect whereby prior exposure alters the response to a subsequent disturbance. Using a 23-species bacterial community exposed to sequential ampicillin pulses, we examined two dimensions of priming. The ecological dimension was manipulated by exposing assembled communities to an antibiotic pre-pulse that could alter species composition before a common high-dose pulse. The evolutionary dimension was manipulated by experimentally evolving individual species for increased ampicillin resistance before community assembly. Community-level pre-pulse exposure shifted composition toward resistant taxa before the main disturbance, reducing subsequent compositional change. Prior resistance evolution had the strongest effect on community dynamics, buffering compositional change during the main pulse, relaxing subsequent selection, and altering community-wide gene expression. This buffering arose from both increased resistance within species and accelerated ampicillin detoxification by a dominant degrader, which transiently reduced antibiotic exposure and promoted the persistence of non-degrading taxa. However, greater resistance did not improve recovery. Because resistance was coupled to competitive dominance, diversity after disturbance remained similar to or lower than in ancestral communities, whereas dominant taxa became further enriched. Together, our results show that antibiotic exposure history reshapes microbial disturbance responses through interacting eco-evolutionary and environmental feedbacks. These feedbacks increase resistance to recurrent disturbance but can constrain recovery, revealing a trade-off between resistance and diversity restoration.

## Introduction

Understanding how microbial communities respond to disturbances is fundamental for medicine, biotechnology, and ecosystem management [1–3]. Community responses are typically attributed to ecological interactions among species and evolutionary changes within them [4], yet the abiotic environment can shift dynamically during disturbance and feed back on both. Although resource-consumer theory captures environmental feedbacks on ecological and evolutionary dynamics [5, 6], empirical work explicitly integrating all three processes remains limited, particularly for microbial communities comprising more than two species.

Such communities underpin essential ecosystem functions but are sensitive to anthropogenic stressors such as antibiotics, heavy metals, and agrochemicals [4, 7–10]. The community response to disturbance can be decomposed into resistance, the capacity to remain unaltered during disturbance, and recovery (or resilience), the capacity to return toward the pre-disturbance state [4]. Because microbes evolve rapidly on ecological timescales owing to their large population sizes and short generation times, both ecological species sorting and rapid evolutionary change can contribute to these responses.

Prior exposure to a stressor can alter the response to a subsequent disturbance, a history-dependent phenomenon commonly termed priming [4, 11]. Priming describes the effect of exposure history and can arise through several, potentially interacting mechanisms. Physiological priming can involve changes in cellular state or gene regulation, ecological priming can arise through changes in species composition, and evolutionary priming can arise through heritable changes within populations. Here, we focus on the ecological and evolutionary dimensions of priming. At the ecological level, an initial antibiotic exposure can filter susceptible taxa and enrich resistant ones, thereby changing how the community responds to a later disturbance. At the evolutionary level, prior antibiotic selection can increase resistance within taxa, further altering the subsequent community response [11, 12]. These dimensions are not mutually exclusive and may interact within the same community.

Recovery will depend on the relationship between antibiotic resistance and competitive ability among the taxa that persist through disturbance. If resistant taxa are also strong competitors, diversity may remain durably reduced; if resistance carries fitness costs, previously suppressed taxa may re-establish. Despite these predictions, experiments that disentangle the ecological, evolutionary, and environmental processes underlying priming across both disturbance and recovery phases remain scarce [11, 12].

One mechanism capable of coupling environmental change to community dynamics during antibiotic disturbance is enzymatic antibiotic degradation. β-lactamases, which hydrolyse β-lactam antibiotics such as ampicillin, can act as public goods by reducing antibiotic concentrations in the shared environment [10, 13, 14]. Declining concentrations may extend the conditions under which less resistant taxa persist [15], restructure competitive hierarchies [15], and reduce the benefit of resistance [16], thereby shaping subsequent recovery dynamics. Moreover, resistance mechanisms and degradation capacity can evolve rapidly under antibiotic selection [17, 18], and resistance evolution in non-degraders may erode exposure protection [19]. Species interactions can further modulate these evolutionary trajectories [20]. Antibiotic degradation therefore provides a tractable context in which ecological sorting, rapid evolution, and environmental transformation interact through coupled feedbacks.

Our previous work using a 23-species synthetic bacterial community showed that short antibiotic pulses produce highly repeatable community trajectories largely explained by intrinsic species traits, namely growth rate and antibiotic susceptibility [21]. We have also shown that disturbance history shapes subsequent responses through both ecological and evolutionary dynamics [12, 22]. However, whether community-driven modification of the antibiotic exposure environment feeds back on these eco-evolutionary processes has not been explicitly tested.

To contrast species-level evolutionary priming with community-level pre-pulse history, we experimentally evolved individual species under escalating ampicillin exposure before community assembly (Fig. 1). We then assembled four community types differing in which species had undergone this prior selection. Assembled communities subsequently received a pre-pulse of 0, 5, 50, or 500 μg ml^−1^ ampicillin before a common 500 μg ml^−1^ main pulse. The pre-pulse allowed exposure history to alter the later response through changes in community composition and through any evolutionary changes occurring during community propagation.

**Figure 1:**
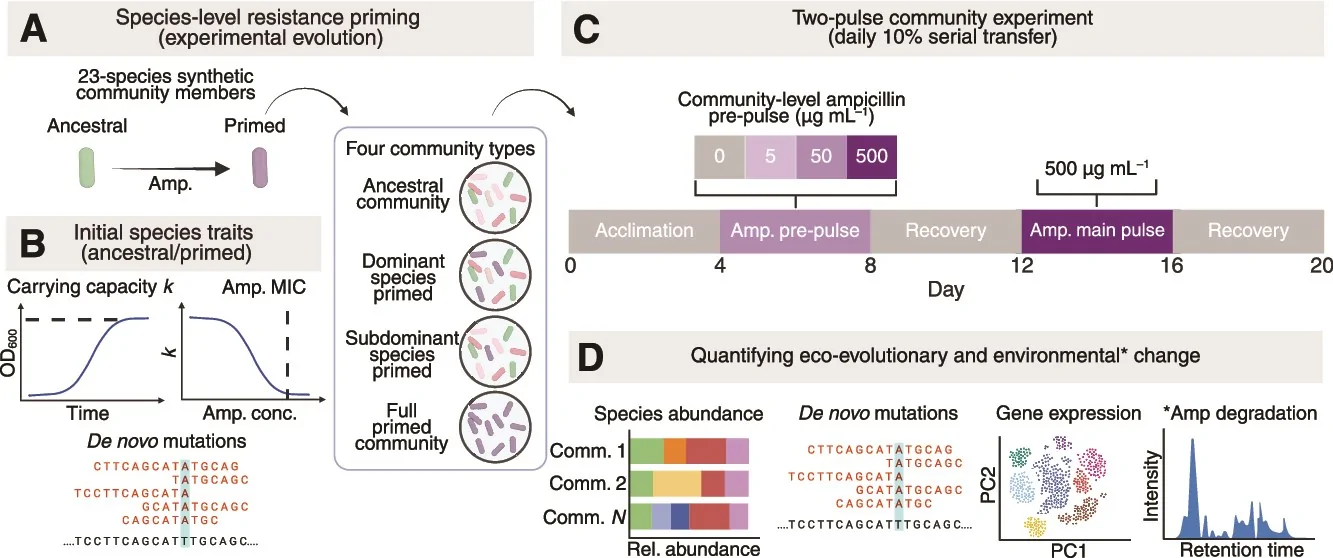
Experimental design. (A) Each of 23 bacterial species was experimentally resistance-primed under increasing ampicillin exposure, generating paired ancestral and primed strains. (B) Initial carrying capacity (*k*), ampicillin MIC, and mutations acquired during resistance priming were characterized, and strains were assembled into four community types: ancestral, dominant-species (*A. caviae*) primed, subdominant-species (*P. chlororaphis*) primed, and fully primed. (C) Communities underwent a 20-day experiment with daily 10% serial transfer, consisting of 4-day epochs: acclimation, a community-level ampicillin pre-pulse (0, 5, 50, or 500 μg ml^−1^), recovery, a common 500 μg ml^−1^ main pulse, and final recovery. (D) During the 20-day experiment, species abundance, de novo mutations, gene expression, and ampicillin degradation were quantified.

We predicted that prior resistance evolution within species would increase community resistance and reduce the additional influence of community-level pre-pulse priming [11, 12], whereas communities assembled from ancestral strains would depend more strongly on ecological species sorting and rapid evolution during the pre-pulse [3, 21, 23, 24]. We further predicted that priming could alter antibiotic degradation dynamics through ecological or evolutionary changes, thereby modifying antibiotic exposure and amplifying community-level protection [15]. By quantifying community composition, functional traits, antibiotic degradation dynamics, and genomic and transcriptional responses, we tested how the ecological and evolutionary dimensions of priming interact with community-driven environmental change to shape microbial resistance and recovery under recurrent antibiotic disturbance.

## Materials and methods

### Model community and culture conditions

The study employed a previously established 23-species Gram-negative synthetic bacterial community [12, 21, 25, 26]. Community members, isolated from diverse habitats (soil, water, or host organisms), were selected for their ability to grow in uniform culture conditions and for bioinformatic separability via 16S rRNA marker gene sequences. All species have been phenotypically characterized and whole-genome sequenced [26, 27]. All experiments used Reasoner’s 2A (R2A) liquid medium at 30°C with shaking at 1000 rpm, unless stated otherwise.

### Experimental evolution for evolutionary priming treatment

To manipulate the evolutionary dimension of priming, all 23 community members were individually subjected to experimental evolution under stepwise-escalating ampicillin exposure before community assembly. This treatment was designed to generate heritable changes in antibiotic resistance (i.e. resistance priming), allowing us to test how prior evolutionary exposure history affected the response of the subsequently assembled communities.

Monocultures were grown in 100% R2A at each species’ IC_50_ ampicillin concentration at 25°C with constant shaking (70 rpm), serially transferred every 96 h using an 8% transfer volume, and exposed to doubling ampicillin concentrations until growth ceased. Resistance priming comprised nine transfer cycles (i.e. ampicillin doublings), except for one species, *Paracoccus denitrificans* HAMBI 2443, which was able to grow only for five cycles.

Assuming complete regrowth from the 8% inoculum to the pre-transfer population density, each cycle corresponded to 3.6 generations. Five and nine transfer cycles therefore corresponded to approximately 18 and 33 generations, respectively. Single clones were isolated from the highest ampicillin concentration supporting growth and freeze-stored with glycerol at −80°C. To characterize genetic changes associated with resistance priming, one resistance-primed founder clone from each of ten selected species was whole-genome sequenced. These species were chosen because they represented abundant members of the synthetic community and exhibited contrasting resistance and growth responses following priming. The sequenced clones were the same founder clones subsequently used to initiate the community experiment.

### Estimating species growth and ampicillin susceptibility

Growth characteristics and ampicillin susceptibility were determined from growth curve measurements (BioTek LogPhase 600 Microbiology Reader) across 23 ampicillin concentrations (0.27–2000 μg ml^−1^, 2,3 dilution series) in two technical replicates per ancestral or resistance-primed clone of each species (100% R2A, 150 μl, 30°C, 800 rpm, optical density at 600 nm every 10 min for 48 h). Carrying capacity (*k*) was extracted from fitted growth curves; *k* values below 0.1 or above 1.3 were excluded. The minimum inhibitory concentration (MIC) was the lowest ampicillin concentration at which no growth was observed, and MIC values were log_10_-transformed for linear modeling. For comparisons of *k* between ancestral and resistance-primed strains, *k* values were taken from the antibiotic-free growth condition (0 μg ml^−1^ ampicillin). This allowed fitness effects of resistance priming to be quantified in the absence of antibiotic selection.

### Two-pulse community experiment

Four 23-species communities were assembled differing only in resistance priming history: (i) ancestral community (all ancestral clones), (ii) dominant species *Aeromonas caviae* primed, (iii) subdominant species *Pseudomonas chlororaphis* primed, and (iv) full primed community (all species primed). *Aeromonas caviae* was chosen as the consistent dominant community member under antibiotic-free conditions [12, 21]; *P. chlororaphis* was chosen as an abundant member exhibiting the strongest resistance-associated carrying capacity cost after priming (Fig. 2A).

**Figure 2:**
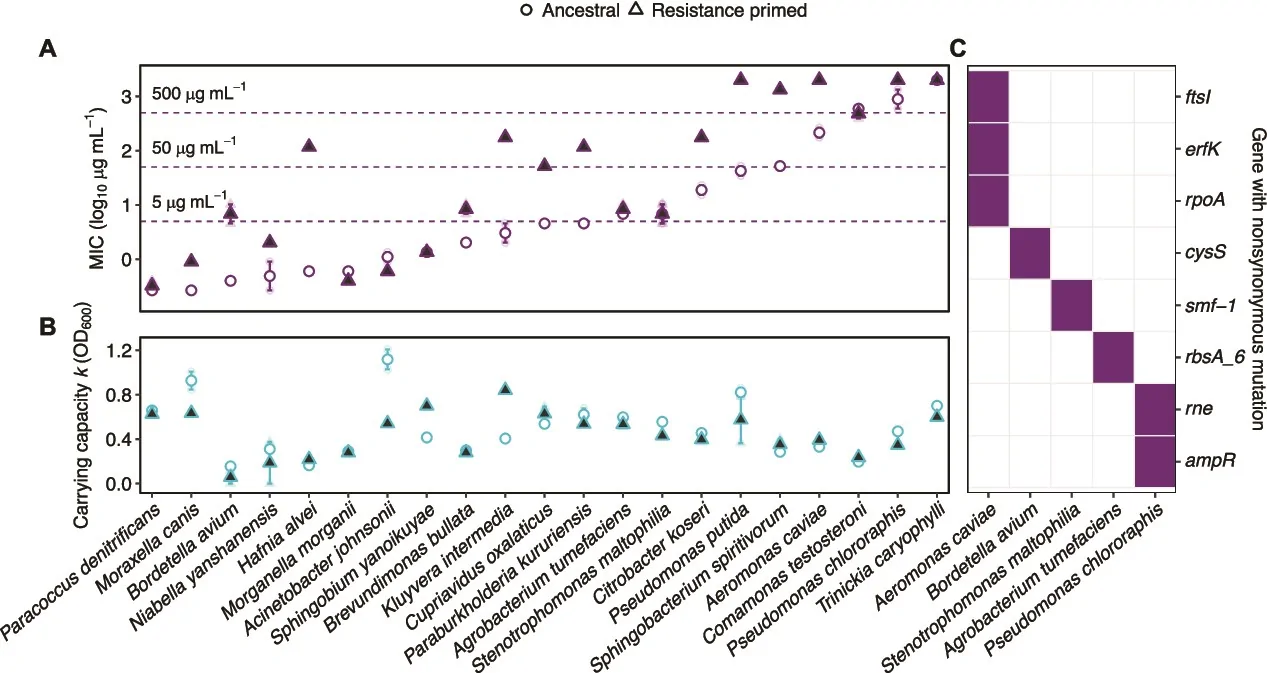
Trait and genomic evolution of resistance-primed species. (A) Log_10_-transformed ampicillin MIC (purple) and (B) antibiotic-free carrying capacity (*k*; OD_600_; turquoise) of synthetic community members before (ancestral isolates; circles) and after resistance priming (triangles). Faint symbols show *n* = 2 technical replicate measurements per clone; large symbols and error bars show the mean ± s.e.m. Dashed lines in A mark the pre-pulse ampicillin concentrations of 5, 50, and 500 μg ml^−1^ and are community exposure references, not MIC thresholds. Data are shown for 21 of 23 community members (ancestral and resistance clones each); two species, *Chitinophaga sancti* and *Microvirga lotononidis*, were omitted due to poor growth. (C) Genes containing nonsynonymous mutations in resistance-primed clones from abundant community members. Ten resistance-primed clones were whole-genome sequenced, one per species; five contained at least one nonsynonymous mutation.

A full-factorial serial passage experiment crossed four resistance priming histories with four community-level pre-pulse ampicillin concentrations (0, 5, 50, or 500 μg ml^−1^) in four biological replicates each, yielding 64 communities in total. Ampicillin concentrations refer to final concentrations in the 2.5 ml culture volume. Ampicillin stock solutions of 1, 10, and 100 mg ml^−1^ were prepared in sterile water, sterile-filtered through a 0.2 μm PES membrane, and stored at −20°C. A constant volume of 300 μl of the appropriate stock solution was added to each 60 ml medium batch to obtain final concentrations of 5, 50, or 500 μg ml^−1^, respectively; the antibiotic-free control received the same volume of sterile water. Fresh treatment medium was prepared at each transfer. The non-zero pre-pulse concentrations were selected to span the measured MIC distribution of the 23 community members, from concentrations affecting primarily the most susceptible taxa to concentrations approaching or exceeding the MICs of many species (Supplementary Table S1). The highest concentration, 500 μg ml^−1^, was also used as the standardized high-intensity main pulse.

Communities were passaged through five 96 h epochs: acclimation, ampicillin pre-pulse, intermediate recovery, main pulse (500 μg ml^−1^ for all communities regardless of pre-pulse level), and final recovery, with serial transfer every 24 h (10% transfer volume) into 48-well plates (2.5 ml total volume). Cell density was estimated by flow cytometry (Guava easyCyte 5 HT HP, Cytek) every 24 h. Samples for 16S rRNA amplicon sequencing, liquid chromatography–mass spectrometry (LC–MS) analysis of ampicillin concentration, and freeze-storage were collected every 96 h (end of each epoch). RNA-seq and metagenomic samples were collected after the main pulse (Day 16) and at experiment end (Day 20), respectively. Communities were assembled from monoculture stocks equalized to 5 × 10^6^ cells ml^−1^ in M9 salt solution by flow cytometry-estimated density, combining 250 μl per species.

### Mass spectrometric analysis of ampicillin degradation

Ampicillin degradation was assessed by monoculture LC–MS assays on four species that grew or persisted during the main community pulse (*A. caviae, Bordetella avium, Comamonas testosteroni*, and *P. chlororaphis*) and by leave-one-out community experiments that sequentially removed each of the three identified degraders, or all three simultaneously, from ancestral or full primed community backgrounds. Cultures were inoculated at 500 μg ml^−1^ ampicillin in 1 ml R2A; cell-free supernatants were collected at 6, 12, and 24 h by centrifugation and 0.2 μm filtration and stored at −20°C.

Ampicillin concentrations were quantified by Turku Metabolomics Center using an Agilent 6460 Triple Quadrupole LC–MS system (Agilent Technologies, Palo Alto, CA, USA) operated in positive multiple reaction monitoring (MRM) mode. Chromatographic separation was performed on an Acquity UPLC HSS T3 column (2.1 × 100 mm, 1.8 μm; Waters Corporation, Milford, MA, USA). The mobile phases consisted of 0.1% formic acid in water (A) and 0.1% formic acid in acetonitrile (B) at a flow rate of 0.45 ml min^−1^. The gradient program was: 0 min, 5% B; 2.0 min, 35% B; 2.5 min, 90% B; 3.0 min, 90% B; 3.1 min, 5% B; and 4.5 min, 5% B. Samples were prepared using deuterated ampicillin (ampicillin-d5) as an internal standard. Additional source conditions were gas temperature 275°C, gas flow 8 l min^−1^, sheath gas temperature 300°C, sheath gas flow 12 l min^−1^, capillary voltage 3500 V, and nozzle voltage 500 V.

### DNA extraction and sequencing

Genomic DNA was extracted using the DNeasy 96 Blood & Tissue Kit (Qiagen) following the manufacturer’s protocol. 16S rRNA amplicon libraries (V3 region; TaggiMatrix system [28, 29]) were sequenced on a MiSeq System (Illumina) using 2 × 150-bp reads. Whole-genome sequencing (WGS) of resistance-primed clones from 10 dominant species was performed using Illumina DNA Prep kits and a NovaSeq X Plus System (Illumina) with 2 × 151 bp reads at SeqCenter (https://www.seqcenter.com). Metagenome and RNA-seq libraries were sequenced on a NovaSeq 6000 System (Illumina) at the Finnish Functional Genomics Centre (FFGC). RNA was extracted using a Monarch Total RNA Miniprep Kit (New England Biolabs), with bead-beating disruption in a TissueLyser II (Qiagen) and on-column DNase treatment.

### Sequence data processing

16S rRNA amplicon data were processed by demultiplexing, quality trimming, paired-end merging, and mapping to community reference sequences [30], with normalization by 16S rRNA gene copy number per species. WGS and metagenomic variants were identified by aligning reads to a custom HAMBI reference genome database (BWA-MEM [31]), followed by GATK Mutect2 [32] variant calling, hard filtering of single nucleotide polymorphisms (SNPs) and insertions/deletions (INDELs), SnpEff [33] annotation, and retention of protein-altering changes only [12]. Copy number variants (CNVs) were detected using CNproScan v0.999 with GC content and mappability normalization [34]; recurrent duplications (≥2 independent communities) were retained. RNA-seq reads were pseudoaligned using Salmon v1.10.1 [35] via the nf-core rnaseq pipeline v3.14.0 [36], with community-composition-based normalization of expression values [25]; differential expression was identified using DESeq2 v1.44.0 [37] (false discovery rate [FDR] < 0.05, |log2 fold change| > 1). Full bioinformatics parameters, quality filters, and variant calling thresholds are provided in the Supplementary Methods.

### Statistical analysis

All analyses were conducted in R v4.4.2 [38]. Community composition was analyzed using Bray–Curtis dissimilarity and permutational multivariate analysis of variance (PERMANOVA; adonis2 in vegan [39]; 999 permutations, type = “by terms”). Temporal instability was quantified as trajectory roughness (cumulative |ΔBray-Curtis dissimilarity| across Days 4–20).

Community-level traits were computed as scalar products between species phenotypes and relative abundance in the community (*f*) with generalized least squares (GLS) models (nlme [92]; first-order autoregressive autocorrelation structure). Trait values for the ancestral or resistance primed species were used depending on which species was present in the corresponding community treatment. For a treatment panel, at Day *t*:


$${MIC}_{comm}(t)=\sum_i{f}_i(t)\cdotp \mathit{\ln}\left({MIC}_i\right)$$

$${k}_{comm}(t)=\sum_i{f}_i(t)\cdotp{k}_i$$

The effects of experimental treatments on cell density, species richness, Shannon Index, community MIC, community carrying capacity (*k*), and ampicillin degradation dynamics were analyzed using GLS models (nlme [40]; first-order autoregressive autocorrelation structure). Species MIC and *k* were compared using ANOVA with species × priming interaction terms. Ampicillin degradation kinetics were analyzed using log-rank tests and restricted mean survival time (RMST) to 24 h. Pairwise contrasts were performed with emmeans [41], and model selection with stepAIC in MASS [42]. MIC and carrying capacity (*k*) of ancestral vs. resistance primed species were assessed with linear regression, and mutation counts with Poisson regression. For species compositional ordination plots, t-SNE was applied to the species-by-sample abundance matrix using Rtsne [43, 44] (perplexity = 15, θ = 0.5, dims = 2, PCA preprocessing enabled). Portions of the analysis code were refactored with the assistance of ChatGPT (GPT-5.2 Thinking; OpenAI, accessed July 2026); the tool was also used for minor language editing. All code and outputs were verified by the authors.

## Results

### Resistance priming produced species-specific resistance and growth changes

Resistance priming broadly increased MIC levels across the 23 community members, with marked species-specific variation in magnitude (ANOVA on log_10_ MIC: resistance priming *F*_1,46_ = 28.4, *P* < .001; species × priming *F*_22,46_ = 6.7, *P* < .001; Supplementary Tables S1 and S2; Fig. 2A): 15 species exhibited higher MIC after priming. In contrast, 14 primed species showed decreased carrying capacity (*k*) in antibiotic-free conditions (7 species with *k* reduction >0.1 OD_600_; ANOVA: priming *F*_1,46_ = 5.0, *P* = .031; species × priming *F*_22,46_ = 5.3, *P* < .001; Supplementary Table S3; Fig. 2B). A pattern consistent with classical resistance-fitness trade-offs [45, 46] was observed in 10 of 23 species (43.5%), which showed increased MIC accompanied by decreased *k*. However, the directions of MIC and *k* change were not significantly associated across species, reflecting substantial diversity in trait responses (Fisher’s exact test: odds ratio = 1.00, 95% CI 0.068–10.32, *P* = 1.00).

WGS of 10 resistance primed clones representing dominant community members identified fixed nonsynonymous mutations in five species (Fig. 2C) and large structural variants in one species (for full details, see Supplementary Results). In *A. caviae*, mutations occurred in *ftsI* (encoding penicillin-binding protein 3 [PBP3]; involved in peptidoglycan cross-linking and β-lactam resistance [28, 29]) and *erfK* (encoding L,D-transpeptidase enabling alternative peptidoglycan cross-linking associated with β-lactam resistance) [29], together with a mutation in *rpoA* (RNA polymerase complex; linked to β-lactamase-independent high-level resistance [30]). *Sphingobacterium spiritivorum* showed a 13× amplification of a genomic block containing the β-lactamase gene *blaB* [31]. *Pseudomonas chlororaphis*, which exhibited the strongest *k* decrease after priming, carried mutations in *rne* [35] (potentially reducing OmpA porin abundance and conferring resistance via reduced permeability [36]) and *ampR* [37] (a regulator of AmpC β-lactamase production). These and additional species-specific genomic findings are reported in the Supplementary Results. Together, phenotypic and genomic changes generated trait differences expected to shape community dynamics under antibiotic disturbance.

### Priming type determined resistance-recovery dynamics by altering trait distribution

The four communities differing in resistance priming history were subjected to the two-pulse experiment and sampled before and after pulses and recovery periods. Increasing pre-pulse ampicillin shifted community composition toward the post-disturbance state prior to the main pulse, indicating ecological pre-conditioning through species sorting (Figs 3 and 4A). The magnitude of compositional change during the main pulse depended on resistance priming background: ancestral and single-species-primed communities showed pronounced restructuring, whereas full-primed communities exhibited buffered trajectories consistent with community-wide resistance evolution (interaction: *F*_3,56_ = 4.4, *P* = .008; Supplementary Table S4; Figs 3 and 4A).

**Figure 3:**
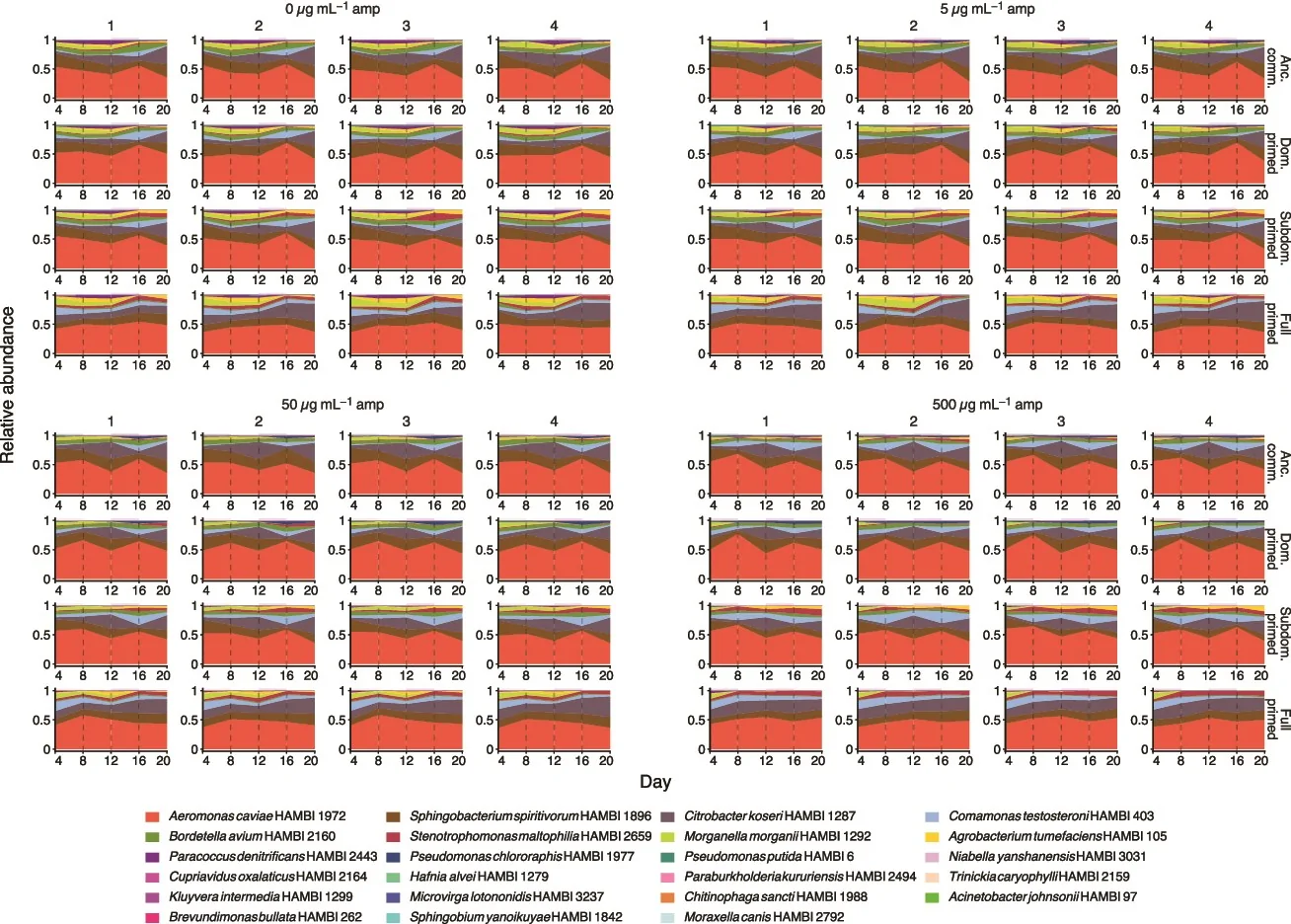
Community composition during the 20-day ampicillin pulse experiment across pre-pulse ampicillin concentrations and resistance-priming backgrounds. Stacked area plots show the relative abundance of individual community members, indicated by color, at Days 4, 8, 12, 16, and 20. The figure is arranged in two vertically stacked blocks: the upper block shows pre-pulse ampicillin concentrations of 0 and 5 μg ml^−1^, and the lower block shows concentrations of 50 and 500 μg ml^−1^. Within each treatment, columns 1–4 represent four independent biological replicates. Rows indicate the resistance-priming background: ancestral community; dominant species (*A. caviae*) primed; subdominant species (*P. chlororaphis*) primed; and full community primed. Each combination of pre-pulse concentration and priming background was represented by *n* = 4 biological replicates, giving 64 communities in total (4 priming backgrounds × 4 pre-pulse treatments × 4 replicates). Vertical dashed lines mark experimental phase boundaries at Days 4, 8, 12, and 16; the pre-pulse phase occurred between Days 4 and 8, and the main pulse phase between Days 12 and 16 (indicated by shade). Species are ordered by overall relative abundance across the experiment.

**Figure 4:**
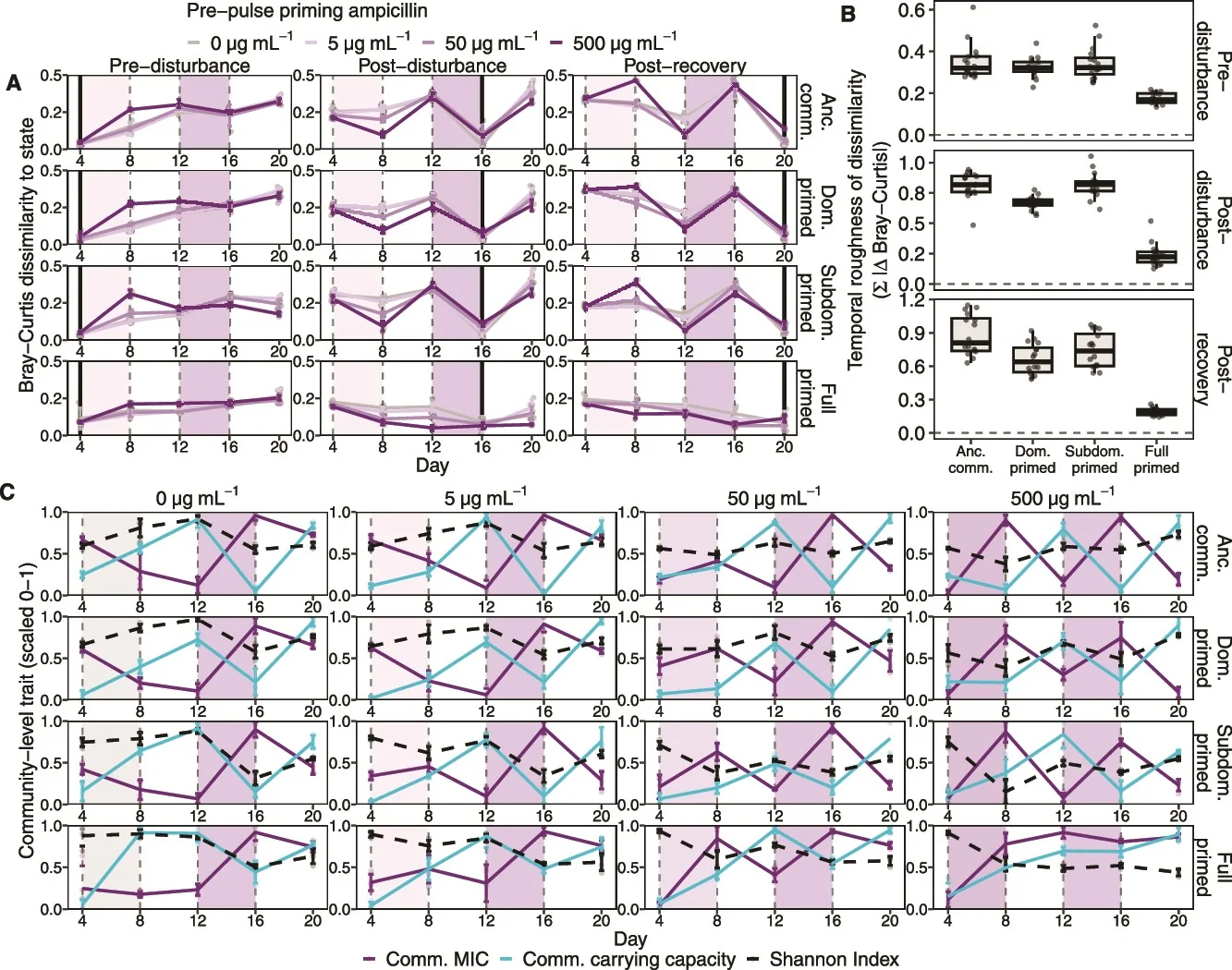
Community dynamics across pre-pulse and resistance priming backgrounds. (A) Bray–Curtis dissimilarity of each community from its resistance-priming-background-specific reference centroid at pre-disturbance (Day 4), post-disturbance (Day 16), and post-recovery (Day 20), indicated by black vertical lines. Points are individual communities (*n* = 4 biological replicates per treatment per time point); solid lines show means with 95% confidence intervals. Shaded regions denote pre-pulse (Days 4–8) and main pulse (Days 12–16) phases. Rows correspond to resistance priming backgrounds. (B) Temporal roughness (Σ|ΔBray–Curtis dissimilarity| across Days 4–20). Box plots show median, interquartile range, and 1.5× interquartile range whiskers. Box plots show temporal roughness values from *n* = 16 communities per resistance-priming background (4 pre-pulse treatments × 4 biological replicates). (C) Community-weighted mean MIC (log-scale, μg ml^−1^), carrying capacity (*k*, OD_600_), and Shannon Index, across time. Traits were min–max scaled within each pre-pulse × background combination. Points represent individual communities (*n* = 4 biological replicates per treatment per time point); solid (MIC, *k*) and dashed (Shannon Index) lines show treatment means with 95% confidence intervals. Columns: pre-pulse ampicillin concentrations (0, 5, 50, 500 μg ml^−**1**^).

These patterns were driven by abundant taxa responding in line with their intrinsic traits. In ancestral communities, *A. caviae* and *C. testosteroni* increased during pulses whereas *Citrobacter koseri, Morganella morganii*, and *S. spiritivorum* declined, consistent with their respective MIC levels (Fig. 2A). In the full primed community, evolved resistance in *C. koseri* and *S. spiritivorum* reduced their decline or promoted their increase, whereas resistance-primed *P. chlororaphis*, despite high MIC, went near-extinct owing to its strong carrying capacity cost (Supplementary Fig. S1). Cell densities were comparable across treatments (Supplementary Fig. S2), confirming relative abundance changes broadly reflected absolute dynamics.

Quantitative community-level analyses confirmed these patterns. Temporal instability was lowest in full-primed communities across all phases (ANOVA, all *P* < .001; Fig. 4B; Supplementary Table S5), including lower post-disturbance roughness (0.19 ± 0.03 vs. 0.67–0.88 in other treatments), demonstrating enhanced ecological resistance to perturbation. Higher pre-pulse intensity shifted communities toward higher community MIC and lower *k* prior to the main pulse (*F*_1,280_ = 103.0, *P* < .001 and *F*_1,280_ = 12.2, *P* < .001, respectively; Supplementary Tables S6 and S7), and stronger pre-pulses advanced community proximity to the final post-recovery state already during intermediate recovery (*F*_1,56_ = 51.6, *P* < .001; Supplementary Table S8), indicating that pre-pulse priming effectively advanced recovery dynamics in time. These trait shifts were largely independent of resistance priming background.

Resistance priming came at a recovery cost. In full-primed communities, increasing pre-pulse intensity reduced species richness (*F*_1,307_ = 40.2, *P* < .001; Supplementary Fig. S3; Supplementary Table S9) and limited recovery of Shannon Index (*F*_3,295_ = 26.7, *P* < .001; Fig. 4C; Supplementary Table S10). These effects arose from dominance of taxa such as *C. koseri* and *S. spiritivorum*, which had evolved moderate to high resistance with low fitness cost. Thus, resistance priming buffered communities during disturbance but constrained recovery by reinforcing competitive dominance among resistant taxa.

## Environmental feedbacks: resistance priming of dominant degrader accelerated ampicillin breakdown and protected non-degraders

Ampicillin concentrations declined to near-zero within each 24 h transfer cycle across all treatments, with slightly faster degradation at higher pre-pulse levels (pre-pulse × day: *F*_1,304_ = 17.5, *P* < .001; Supplementary Fig. S4; Supplementary Table S11), consistent with ecological enrichment of degraders. Because community-level degradation was nearly complete within each cycle, we focused on within-cycle dynamics to identify key degraders and assess the effects of resistance priming on detoxification rate.

Monoculture LC–MS assays identified *A. caviae, C. testosteroni*, and *P. chlororaphis* as rapid ampicillin degraders (Fig. 5A). Resistance priming specifically accelerated ampicillin degradation in *A. caviae*: primed clones reached below 10 μg ml^−1^ by 6 h rather than 12 h (Wilcoxon *W* = 0, *P* = .013), with higher biomass at 6 h (OD_600_: 0.23 vs. 0.04; *P* < .001). Degradation timing correlated negatively with growth rate at 6 h (Spearman’s *ρ* = −0.88, *P* = .021), indicating that accelerated detoxification arose from improved growth under antibiotic stress rather than intrinsically enhanced enzymatic capacity. By contrast, resistance priming did not shift degradation timing in *C. testosteroni*, whereas primed *P. chlororaphis* showed reduced early biomass consistent with its growth cost rather than any improvement in detoxification.

**Figure 5:**
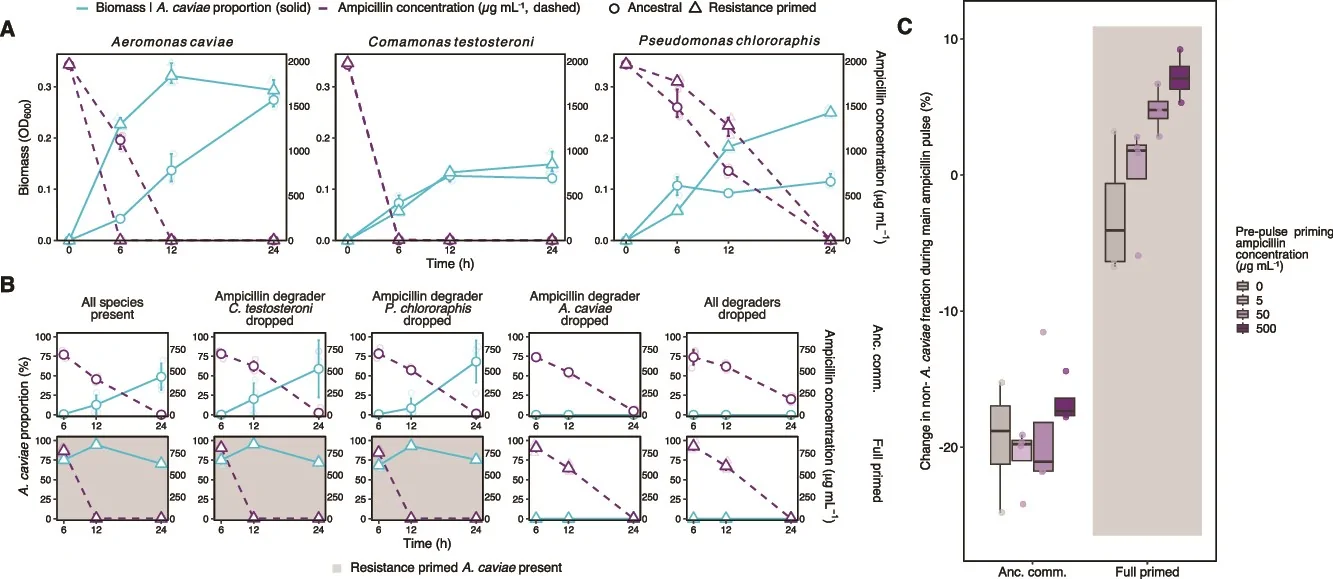
*Aeromonas caviae* dynamics and community responses to ampicillin. Turquoise solid lines show bacterial measurements; purple dashed lines show ampicillin concentration (*C_amp_*). Hollow circles and triangles denote ancestral and resistance-primed bacteria or communities, respectively. (A) Biomass (OD_600_, left axis) and *C_amp_* (μg ml^−1^, right axis) over 24 h in monocultures of *A. caviae, C. testosteroni*, and *P. chlororaphis*. Small faint symbols are individual technical replicates; large symbols and lines show means, with 95% confidence intervals. For each species and priming state, *n* = 3 technical replicate monocultures. (B) *Aeromonas caviae* relative abundance (left axis) and C_amp_ (right axis) at 6, 12, and 24 h in the leave-one-out experiment. Small faint symbols are individual biological replicate communities; large symbols and lines show means with 95% confidence intervals. Each community-background × leave-one-out treatment comprised *n* = 4 independent biological replicates. Rows show ancestral and full resistance-primed communities; columns show all species present, *C. testosteroni* removed, *P. chlororaphis* removed, *A. caviae* removed, or all three degraders removed. Gray shading marks treatments containing resistance-primed *A. caviae*. (C) Change in the non-*A. caviae* fraction during the main pulse in the 20-day experiment, $\Delta f={f}_{\mathrm{day}\;16}-{f}_{\mathrm{day}\;12}$. Points are individual communities (*n* = 4 biological replicates per resistance-priming background × pre-pulse treatment); box plots summarize each group at pre-pulse *C_amp_* levels of 0, 5, 50, or 500 μg ml^−1^. Center lines are medians, boxes span the interquartile range, and whiskers extend to 1.5 times that range. Positive values indicate an increase in taxa other than *A. caviae*; gray shading marks the full resistance-primed community.

To determine how degrader identity and resistance priming shaped community-level detoxification, we performed a 24 h leave-one-out experiment removing individual or all degraders from ancestral or full-primed community backgrounds (Fig. 5B; Supplementary Fig. S5). All degrader combinations produced near-complete ampicillin degradation within 24 h. Moderate ampicillin decline also occurred in the absence of all three focal degraders, suggesting that additional community members may possess limited degradation capacity. Whenever *A. caviae* was present, ampicillin declined significantly earlier in resistance primed than ancestral communities (log-rank *χ*^2^ = 23.00, *P* < .001; ΔRMST = −12 h). Ancestral communities took the full 24 h culture cycle to degrade ampicillin, and *A. caviae* relative abundance increased throughout this period. In full-primed communities, after degradation (12 h), *A. caviae* relative abundance declined and the non-*A. caviae* fraction increased, indicating that accelerated detoxification transiently released competitors from antibiotic stress even though *A. caviae* maintained dominance.

In line with these results, in the 20-day community experiment, non-*A. caviae* taxa declined during the main pulse in ancestral communities but were maintained or increased in full-primed communities (resistance priming effect: *F*_3,48_ = 179.2, *P* < .001; Supplementary Table S12; Fig. 5C). Increasing pre-pulse priming further increased the non-*A. caviae* fraction across backgrounds (*F*_3,48_ = 7.7, *P* < .001), consistent with prior ecological enrichment of resistant taxa. Together, these results show that resistance priming of the dominant degrader *A. caviae* accelerates environmental detoxification and transiently protects non-degraders, whereas *A. caviae* itself maintains dominance owing to its high resistance, efficient degradation, and absence of a fitness cost of resistance.

### Mutation and gene expression patterns reflect relaxed and altered selection with resistance priming

Community metagenomic sequencing at the end of the 20-day community experiment revealed that both community-level pre-pulse priming and species-level resistance priming shifted de novo mutation profiles (PERMANOVA: pre-pulse *F*_1,63_ = 2.1, *R*^2^ = 0.03, *P* = .023; resistance priming *F*_3,63_ = 3.7, *R*^2^ = 0.15, *P* < .001; interaction *F*_3,63_ = 1.6, *R*^2^ = 0.06, *P* = .030; Supplementary Tables S13 and S14; Fig. 6A and B). The effect was dominated by species-level resistance priming: full-primed communities differed significantly from all others in mutation profiles (all post-hoc *P* < .001), whereas other backgrounds were mutually similar (all post-hoc *P* > 0.30).

**Figure 6:**
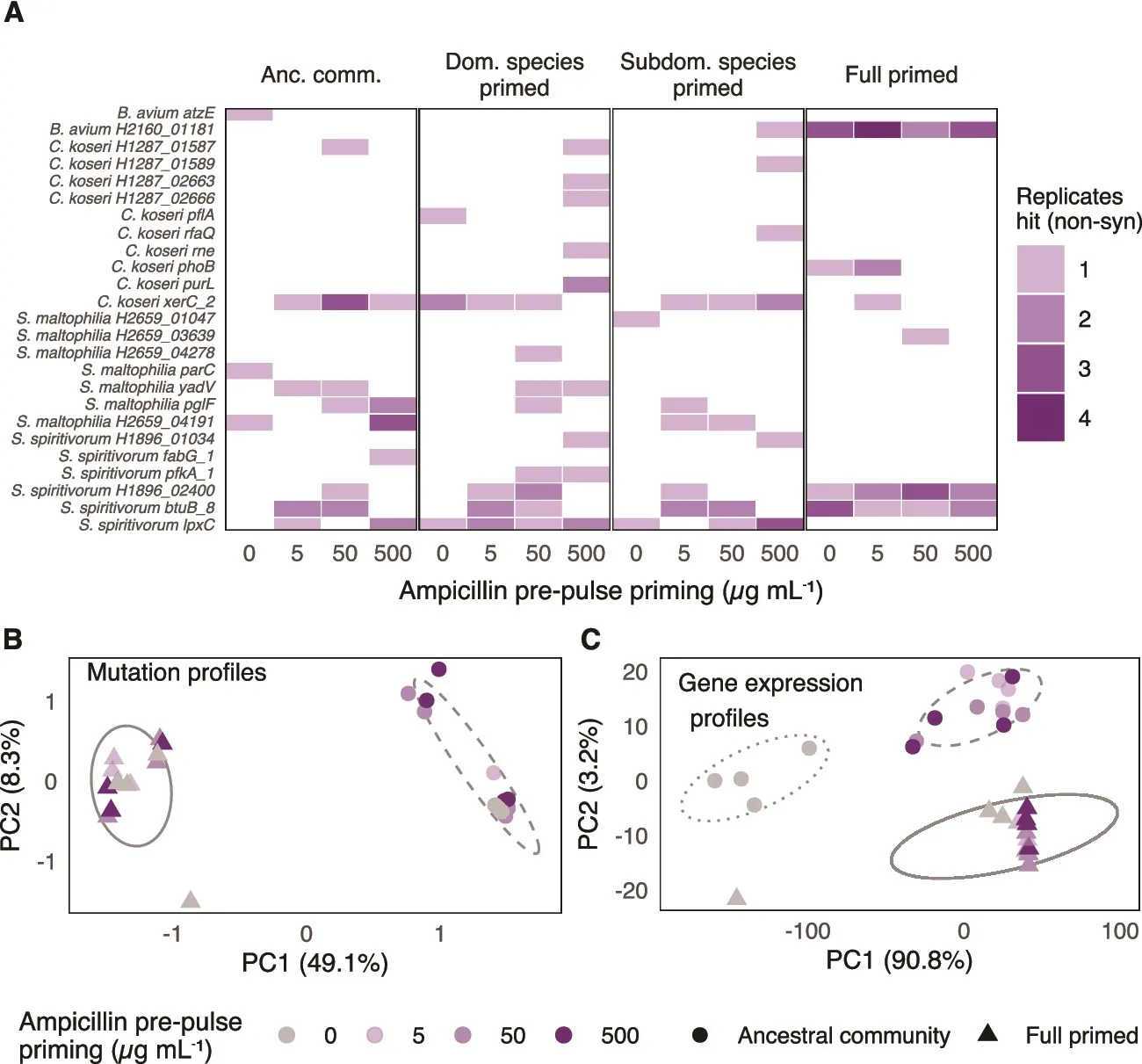
Effect of priming on de novo mutations and gene expression. (A) Heatmap of high-confidence, recurrent (present in more than one independent community) nonsynonymous de novo mutations detected at the experimental endpoint in 64 community metagenomes (4 resistance-priming backgrounds × 4 pre-pulse treatments × 4 biological replicates) across pre-pulse levels (*x*-axis) and resistance priming backgrounds (columns). Tile shade indicates the number of independent replicate communities (1–4) in which the mutation was observed; gene labels include species names. (B) Principal component analysis (PCA) of mutation profiles for ancestral and full-primed communities (*n* = 16 each), with 68% confidence ellipses shown for each group. (C) PCA of gene expression profiles after main ampicillin pulse, normalized by species relative abundances (*n* = 16 biological replicate communities per resistance-priming background), with 68% confidence ellipses for ancestral and full-primed communities at 5–500 μg ml^−1^ pre-pulse, and ancestral communities at 0 μg ml^−1^ pre-pulse.

These differences reflected shifts in mutation targets rather than increased variance (dispersion analysis *F*_3,60_ = 1.1, *P* = .359). For example, in *C. koseri*, mutations recurred at the *xerC* locus (site-specific recombinase) in ancestral and single-species-primed communities but at *phoB* in full-primed communities; in *S. spiritivorum, btuB* was the only recurrent target in full-primed communities but co-occurred with *lpxC* in other backgrounds. Full-primed communities had on average half the number of mutation targets compared with other backgrounds, with more recurrent mutations at the same loci. Similarly, gene duplications (e.g. *infC* in *C. testosteroni*; *xerC* in *C. koseri* and *B. avium*) occurred predominantly in non-full-primed communities (Supplementary results; Supplementary Fig. S6). These results are consistent with reduced selection pressure arising from prior resistance mutations and narrowing of future evolutionary pathways. Moreover, many targets differed from those observed during monoculture resistance priming, in line with prior work showing that the community context fundamentally alters mutation targets under positive selection [20, 47–50].

Gene expression profiles (RNA-seq) after the main antibiotic pulse, normalized by species relative abundances, differed significantly between ancestral and full-primed communities (PC1: *r* = 0.57, *P* < .001, FDR-adjusted *P* = .001; PC2: *r* = −0.71, *P* < .001, FDR-adjusted *P* < .001; Fig. 6C), reflecting a distinct community transcriptional state associated with prior resistance evolution. Within ancestral communities, pre-pulse priming had a marginally significant transcriptional effect primarily driven by an antibiotic-free versus antibiotic-exposed contrast (pre-pulse level: PC2 *r* = 0.50, *P* = .047, FDR = 0.096). Differentially expressed genes were distributed across treatments without clustering into specific functional pathways, consistent with broad transcriptional adjustments driven by global gene expression changes linked to resistance mutations [51–53] rather than targeted pathway-level responses.

## Discussion

Disturbance responses in microbial communities are frequently interpreted through ecological species sorting or evolutionary change alone [4, 11, 12, 21]. Our results suggest that, in systems where antibiotic degradation changes the stress environment, ecological and evolutionary responses are best interpreted together with environmental transformation. By manipulating community-level pre-pulse history and species-level resistance history, we identified distinct contributions of these dimensions and a resistance-recovery trade-off associated with altered antibiotic degradation dynamics (Fig. 7).

**Figure 7:**
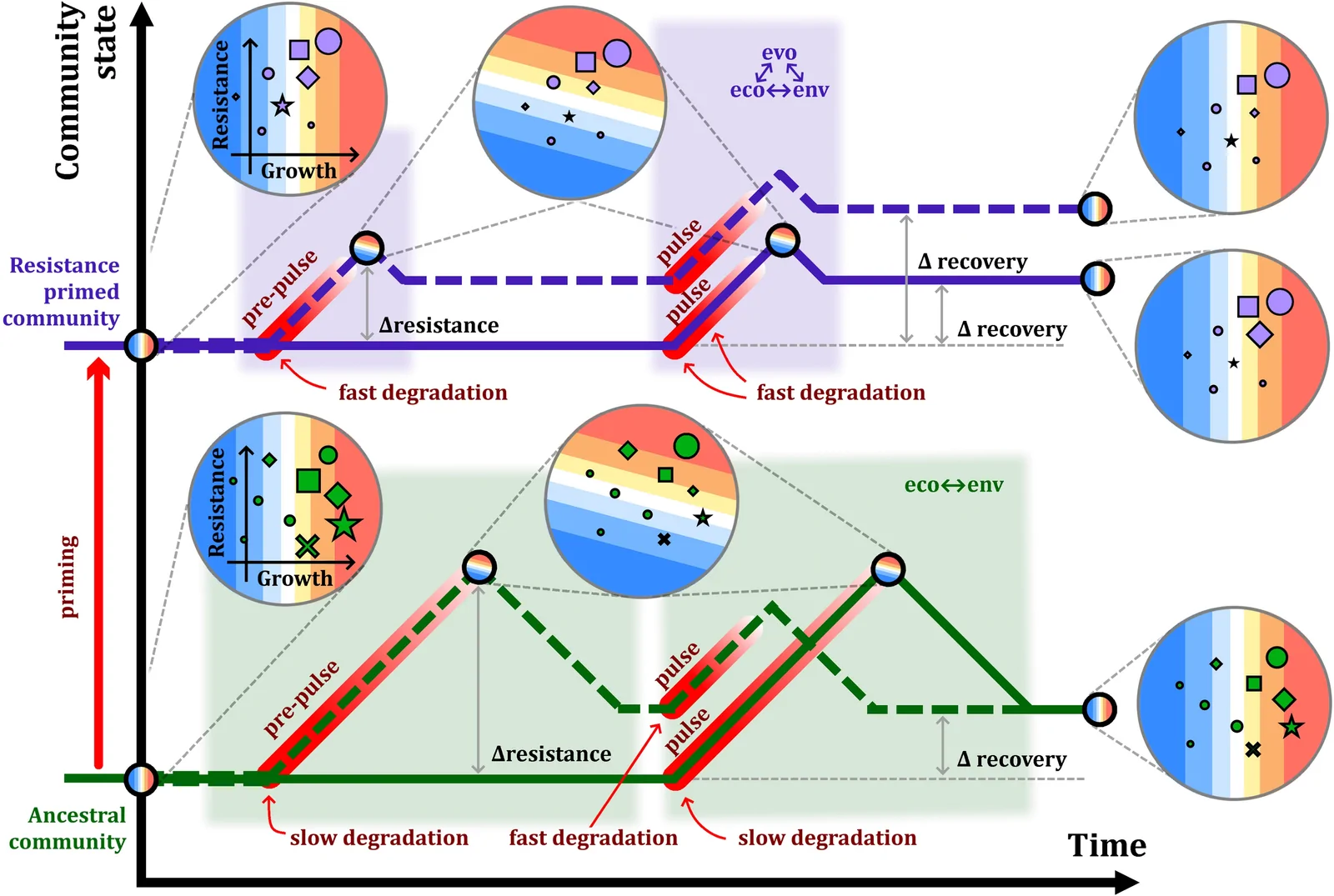
Eco-evolutionary-environmental feedbacks shape community resistance and recovery under recurrent antibiotic disturbance. Green and purple trajectories represent ancestral and resistance-primed communities, respectively; solid and dashed lines indicate communities without and with pre-pulse exposure. The vertical axis represents a general community state, defined as any compositional or functional metric of interest (e.g. relative abundance, diversity, gene expression, or metabolic state), and the horizontal axis represents time; both are conceptual and not to scale. Δresistance represents the magnitude of disturbance-induced change between the pre-disturbance and end-of-pulse community states, whereas Δrecovery represents the residual difference from the pre-disturbance state after recovery; smaller values indicate greater resistance and more complete recovery, respectively. Circular insets show schematic species distributions in growth-resistance trait space, with redder regions indicating favored trait combinations. Ancestral communities undergo stronger species sorting during pulses but recover more fully, whereas resistance-primed communities show greater resistance but more limited recovery, associated with evolved resistance and faster ampicillin degradation.

Consistent with ecological filtering predictions [4, 11, 12], community-level pre-pulse priming accelerated compositional sorting toward resistance-enriched states and reduced the magnitude of change required during the main pulse. The observed effects are more parsimoniously explained by trait-based species sorting than by rapid de novo resistance evolution, extending our earlier finding on the larger contribution of species-trait sorting over de novo evolution in community responses [21] to a two-pulse scenario and aligning with evidence for constrained evolutionary dynamics in community contexts [47, 54]. Pre-pulse priming therefore modified trajectory speed rather than trajectory outcome, effectively advancing in time the sorting that would ultimately occur during the main pulse regardless. This interpretation is supported by the gene expression data: within ancestral communities, pre-pulse priming produced only a weak transcriptional effect distinguishing antibiotic-free from antibiotic-exposed conditions, offering no evidence for broad evolutionary adaptation prior to the main pulse.

In contrast, species-level resistance priming directly altered the evolutionary state of community members before community assembly. When all species were primed, communities showed reduced compositional fluctuation during antibiotic exposure, consistent with evolutionary buffering [11, 12, 55]. Yet this buffering came at a recovery cost: diversity (Shannon Index) declined and dominance structures were reinforced, revealing an ecological cost of evolutionary resistance. Strikingly, ancestral communities recovered as well as or better than full-primed communities despite experiencing stronger compositional change during the pulse. This unexpected result reflects the role of antibiotic degradation in limiting diversity loss in ancestral communities to reversible species sorting rather than local extinction, whereas the coupling of resistance with competitive dominance in primed communities constrained diversity recovery. The two priming modes thus differentially reshaped the balance between resistance and recovery: pre-pulse priming influenced trajectory dynamics but left the final recovered state largely unchanged, whereas resistance priming altered both the disturbance response and the recovery endpoint.

Some observed patterns were not readily accounted for by ecological or evolutionary priming alone and coincided with changes in antibiotic degradation, indicating an additional environmental dimension. Pre-pulse priming modestly accelerated degradation, consistent with compositional enrichment of degraders. Moreover, resistance priming of the dominant degrader *A. caviae* accelerated in-community antibiotic breakdown, producing earlier reductions in effective exposure. The *ftsI* (encoding penicillin-binding protein 3, PBP3) mutation detected in resistance-primed *A. caviae* could have contributed to its faster growth and degradation under ampicillin exposure. PBP3 mutations are known to alter β-lactam susceptibility and cell division dynamics [56, 57], potentially increasing growth under antibiotic stress and enabling populations to reach effective degradation thresholds earlier. Consistently, communities containing primed *A. caviae* reached substantially higher cell densities (Supplementary Fig. S2), supporting altered growth dynamics under ampicillin. However, the contribution of this mutation was not tested directly, and other evolved differences may also have contributed.

Together, these observations are consistent with an emergent eco-evolutionary-environmental feedback (Fig. 7). Resistance evolution in a dominant degrader was associated with faster antibiotic removal and the subsequent expansion of other taxa, suggesting that environmental modification contributed to changes in ecological competition and selection. This is consistent with prior work on the ecological effects of antibiotic degradation, indicating a role for rapid evolution [10, 15]. However, although our monoculture and leave-one-out experiments identify *A. caviae* as a key driver of this feedback, because each link in this proposed feedback was not independently manipulated, the full causal sequence remains to be tested directly. Future pairwise experiments combining ancestral or resistance-primed degraders with susceptible non-degraders could further distinguish detoxification-mediated protection from direct competition and test how these interactions scale with community complexity.

More broadly, these results support the view that microbial disturbance responses can be path-dependent through both altered trait distributions and changes in the stress environment. In antibiotic-exposed microbiomes and other disturbed systems [1, 22, 58–60], such environmentally mediated evolutionary effects may contribute to whether communities stabilize, collapse, or reorganize following recurrent stress.

The resistance-recovery trade-off identified here is not apparent when either property is considered alone and may be amplified in natural communities, where antibiotic degradation commonly contributes to community-level tolerance [13, 15]. This environmental feedback should be strongest when resistance reduces antibiotic exposure for other taxa. If resistance were instead dominated by cell-autonomous mechanisms, such as target modification or increased efflux, ecological sorting and evolutionary priming could still occur, but without comparable detoxification or cross-protection. Selection might then persist longer during each pulse, with community outcomes depending more directly on species-specific resistance, its fitness costs, and competitive ability. Under such conditions, the specific trade-off observed here may be weakened because the recovery of susceptible taxa would no longer benefit from degrader-mediated protection. Future work should test whether this trade-off holds across different degradation efficiencies, antibiotic classes, and levels of community diversity, and whether such eco-evolutionary-environmental feedbacks can be used to steer community composition in microbiome management or bioremediation.

By integrating ecological filtering, evolutionary priming, and environmental transformation within a unified experimental design, our study highlights the value of considering their interactions when interpreting microbial disturbance responses. Disturbance history may alter both community composition and the stress environment encountered upon re-exposure. The eco-evolutionary-environmental framework developed here therefore provides a conceptual basis for investigating how prior stress history contributes to variation in resistance and recovery trajectories.

## Supplementary Material

SI_ISME_final_wrag248

## Data Availability

The 16S rRNA gene amplicon sequencing data from the main microbial community evolution experiment are available in the European Nucleotide Archive under accession PRJEB108471, and whole-genome, metagenome, and RNA-seq data under the accessions PRJEB109096, PRJEB109023, and PRJEB109068, respectively. Pre-processed data for all downstream analyses are available in GitHub: https://github.com/johannescairns/eco-evo-detox-ampicillin.

## Code availability

The code needed to reproduce the downstream analyses for RNA-seq data is available at https://version.helsinki.fi/biodata-analytics-unit/ampicillin-pulse-experiment. The code for reproducing all other downstream analyses and figures is available in GitHub: https://github.com/johannescairns/eco-evo-detox-ampicillin.
